# Short and long term gene expression variation and networking in human proximal tubule cells when exposed to cadmium

**DOI:** 10.1186/1755-8794-6-S1-S2

**Published:** 2013-01-23

**Authors:** Scott H Garrett, Kaitlin Clarke, Donald A Sens, Youping Deng, Seema Somji, Ke K Zhang

**Affiliations:** 1 Department of Pathology, School of Medicine and Health Sciences, University of North Dakota, Grand Forks, ND 58202, USA; 2 Bioinformatics Core, School of Medicine and Health Sciences, University of North Dakota, Grand Forks, ND 58202, USA; 3 Rush University Cancer Center, Chicago, IL 60612, USA

## Abstract

Cadmium (Cd^2+^) is a known nephrotoxin causing tubular necrosis during acute exposure and potentially contributing to renal failure in chronic long-term exposure. To investigate changes in global gene expression elicited by cadmium, an *in-vitro *exposure system was developed from cultures of human renal epithelial cells derived from cortical tissue obtained from nephrectomies. These cultures exhibit many of the qualities of proximal tubule cells. Using these cells, a study was performed to determine the cadmium-induced global gene expression changes after short-term (1 day, 9, 27, and 45 μM) and long-term cadmium exposure (13 days, 4.5, 9, and 27 μM). These studies revealed fundamental differences in the types of genes expressed during each of these time points. The obtained data was further analyzed using regression to identify cadmium toxicity responsive genes. Regression analysis showed 403 genes were induced and 522 genes were repressed by Cd^2+ ^within 1 day, and 366 and 517 genes were induced and repressed, respectively, after 13 days. We developed a gene set enrichment analysis method to identify the cadmium induced pathways that are unique in comparison to traditional approaches. The perturbation of global gene expression by various Cd^2+ ^concentrations and multiple time points enabled us to study the transcriptional dynamics and gene interaction using a mutual information-based network model. The most prominent network module consisted of INHBA, KIF20A, DNAJA4, AKAP12, ZFAND2A, AKR1B10, SCL7A11, and AKR1C1.

## Introduction

Cadmium is a toxic heavy metal that is widely distributed due to industrial pollution such as mining, refining of metals, burning of fossile fuels, battery production and the use of tobacco. The health threats presented by cadmium has been increasingly recognized by government agencies, healthcare providers and the general public [1]. One of the primary target organs of cadmium toxicity is the human kidney where cadmium accumulates and induces a number of adverse events. The proximal tubule is the main site for cadmium accumulation and the site of cadmium induced toxicity. When the levels of cadmium exceed a tolerance level of the intracellular defense systems, such as metallothionein and glutathione, cells become susceptive to the toxic effect of this heavy metal. [2,3]. Under high dose acute exposure, cadmium is known to cause proximal tubule necrosis. On the other hand, a few cohort studies showed chronic low doses of cadmium can also induce proximal tubule toxicity [4,5]. Toxicokinetic models indicated that the half-life of cadmium in humans is between 15 and 20 years [6]. This suggests that low level exposure of cadmium may be harmful to human kidney via the long term accumulation of this metal in proximal tubule. In this study, we investigate the acute and chronic effects of cadmium on human proximal tubule (HPT) cells. The HPT cell culture system developed in our lab has been shown to have important properties that resemble human proximal tubule [7]. The ability of forming dome by confluent cell monolayers implies the HPT cells retain the vectorial transport activity. The cells are mortal and exposure to high concentration of cadmium induces a necrotic mechanism of cell death [8,9]. By exposing HPT cells to various doses of cadmium in either a short period of 1 day or a long period of 13 days, we expect different physiological responses to be elicited which represent the acute and chronic responses to cadmium toxicity. In order to investigate the underlying molecular mechanisms of cell toxicity and compensatory cellular defenses related to cadmium exposure, we profiled the global gene expression in HPT cells exposed to increasing concentrations of this metal during short-term (1 day) and long-term (13 days) exposure.

An initial analysis of the microarray data has previously reported a number of genes that were differentially expressed between cadmium treated and control samples [10]. In this manuscript, we investigated how genes respond to different doses of cadmium and how the gene network is formed in response to cadmium toxicity. The high cost of microarray chips prevents researchers from collecting microarray data with large sample sizes; thereby, limiting the power of statistical analysis. In this study, we only have one sample for each condition-time point, however, the overall experimental design enabled us to investigate the gene expression profiles correlated with cadmium concentration. Furthermore, we developed a gene set enrichment analysis (GSEA) method that can be applied to regression-based tests for individual genes, while traditional GSEA methods focus on two-sample comparison analysis, such as t test.

This study adopted a complicated design by perturbing HPT cells with a variety of doses of cadmium and under various time periods that resulted in variation and dynamics of global gene expression. Therefore, it provides a unique opportunity to investigate the dynamic change of gene expression levels and the network structure of gene interactions in a well-designed experimental environment. A mutual information-based network model [11] was used to investigate the gene networking in response to cadmium toxicity and a network module with a small set of activated genes with high interaction between each other was identified.

## Materials and methods

### Cell culture and total RNA collection

The HPT cell culture system and cadmium treatment has been described previously [7,10]. RNA was purified from triplicate cultures of HPT cells by the RNeasy Mini kit (Qiagen, Valencia, CA). The integrity and quality of RNA was ensured via the ribosomal bands using agarose gel electrophoresis.

### Microarray analysis

Microarray experiment was performed by Genome Explorations Inc. (Memphis, TN) using Affymetrix GeneChip Human Genome U133 Plus 2.0 arrays, which cover over 47,000 human transcripts and variants representing approximately 39,000 of the best characterized human gene transcripts and expressed sequence tags (ests) (Affymetrix, Santa Clara, CA). The scanned images were analyzed using programs resident in GeneChip Operating System v1.4 (GCOS; Affymetrix). The raw data was processed and normalized using MAS 5.0 statistical algorithm that gave detection call regarding signal present or absent for each probe. The correlation of gene expression with cadmium concentrations was assessed using linear regression. The gene signal pathways that were activated by cadmium were identified using gene set enrichment analysis (GSEA) [12]. The R package GSA that was based on standardized statistics was employed for gene set enrichment analysis [13]. The gene list for each pathway was obtained from the Kyoto Encyclopedia of Genes and Genomes (KEGG) http://www.genome.jp/kegg. Cluster analysis was used to group the microarray samples using hierarchical clustering method. The distances between samples were calculated by Pearson dissimilarity and the agglomeration of clusters was performed by Ward linkage method.

### Networks analysis

The relations between genes were defined by mutual information [11]. The resulting adjacent matrix was used for networks analysis. Network modules were identified by hierarchical clustering. The set of genes that have short distances between each other and large distance with other genes was defined as a network module. The most prominent network module was identified for further investigation. All data analyses were performed using R statistical programming language.

## Results

### Gene expression variation in response to acute (1 day) cadmium toxicity

**Figure 1:**
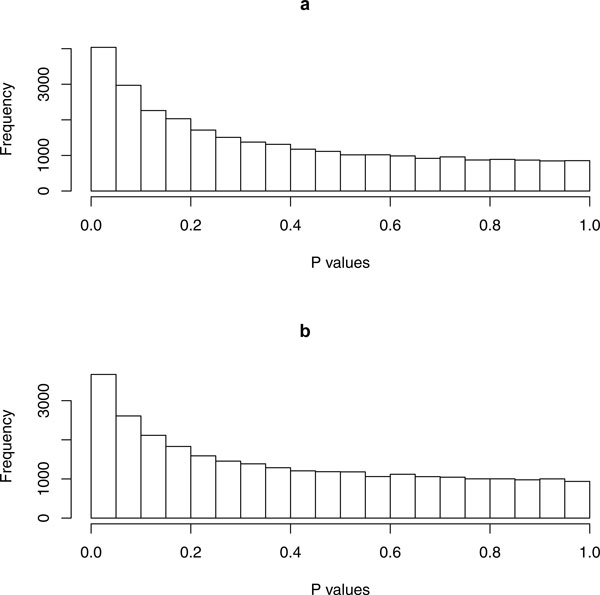
**The distributions of P values in the regression analysis of gene expression versus cadmium concentration**. (a) The histogram of P values for 1 day exposure of cadmium. (b) The histogram of P values for 13 days exposure of cadmium.

The HPT cells were exposed to various levels of concentrations of Cd^2+ ^for a short term of 24 hours. The Cd^2+ ^concentrations were 0, 9, 27, and 45 *μM*. Our hypothesis is that Cd^2+ ^exposure will elicit an acute toxicity response to HPT cells within one day. As reported before, the HPT cells exhibited a stress response during 24 hour exposure to increasing concentrations of Cd^2+ ^and showed visible signs of toxicity at the high dose [10]. The goal of the current study is to discover the genes that are responsive to the short-term exposure of Cd^2+ ^using messenger RNA microarray data. For each Cd^2+ ^concentration, a sample of HPT cells was harvested, total RNA was purified and submitted for microarray analysis. The Affymetrix U133 Plus 2.0 array was used for this analysis. When using MAS 5.0 for testing, we found 28,720 genes that were present in at least one sample. These genes were used for subsequent analysis. In order to identify the genes whose expression levels were dependent on Cd^2+ ^concentrations, we conducted regression analysis for correlating gene expression with concentrations. This resulted in 4,036 genes that have significant P values (< 0.05). However, none of them had a false discovery rate below 0.05 due to the small size of only 4 samples. Figure 1a shows the P value distribution that was tilted to low values implying that many genes had positive responses to acute Cd^2+ ^toxicity. If only a few genes had positive responses, the P values would be evenly distributed. Using a P value cutoff of 0.01, we reported 403 genes that were positively correlated with cadmium concentration (Table 1), and 522 genes that were negatively correlated (Table 2). The positively correlated genes in the top list included heat shock genes like HSP40, HSP70 and HSPBAP1, and ion and anion transporters SLC7A11, SLC16A1, SLC20A2, SLC25A37 and SLC7A1. Both the highest positively and negatively correlated genes contained cell cycle arrest and cell death genes such as G0S2, GSPT1 and BAG5. These activated genes represented a stress response for the acute Cd^2+ ^toxicity.

**Table 1:** Cd^2+ ^induced genes in acute cadmium-exposed human proximal tubule cells (1 day, p value < 0.001)

Probe	Symbol	Gene name	Pr(> |t|)
225061_at	DNAJA4	DnaJ (Hsp40) homolog, subfamily A, member 4	1.16E-05
207528_s_at	SLC7A11	solute carrier family 7, (cationic amino acid transporter, y+ system) member 11	1.66E-05
209774_x_at	CXCL2	chemokine (C-X-C motif) ligand 2	4.83E-05
202744_at	SLC20A2	solute carrier family 20 (phosphate transporter), member 2	5.00E-05
213524_s_at	G0S2	G0/G1 switch 2	7.15E-05
220533_at	NA	NA	7.69E-05
208544_at	ADRA2B	adrenergic, alpha-2B-, receptor	7.89E-05
239211_at	NA	NA	8.93E-05
225339_at	SPAG9	sperm associated antigen 9	0.000113
202235_at	SLC16A1	solute carrier family 16, member 1 (monocarboxylic acid transporter 1)	0.000129
209406_at	BAG2	BCL2-associated athanogene 2	0.000134
214577_at	MAP1B	microtubule-associated protein 1B	0.000147
215404_x_at	FGFR1	fibroblast growth factor receptor 1	0.000153
229724_at	GABRB3	gamma-aminobutyric acid (GABA) A receptor, beta 3	0.000183
215034_s_at	TM4SF1	transmembrane 4 L six family member 1	0.000194
224809_x_at	TINF2	TERF1 (TRF1)-interacting nuclear factor 2	0.000212
212223_at	IDS	iduronate 2-sulfatase	0.000216
223144_s_at	AKIRIN2	akirin 2	0.000226
225267_at	KPNA4	karyopherin alpha 4 (importin alpha 3)	0.000228
204186_s_at	PPID	peptidylprolyl isomerase D	0.000228
200895_s_at	FKBP4	FK506 binding protein 4, 59 kDa	0.000269
226541_at	FBXO30	F-box protein 30	0.000307
204041_at	MAOB	monoamine oxidase B	0.000372
1557915_s_at	GSTO1	glutathione S-transferase omega 1	0.000435
202199_s_at	SRPK1	SFRS protein kinase 1	0.000476
211696_x_at	HBB	hemoglobin, beta	0.000491
224888_at	SELI	selenoprotein I	0.000593
227408_s_at	SNX25	sorting nexin 25	0.000618
222703_s_at	YRDC	yrdC domain containing (E. coli)	0.000654
229355_at	NA	NA	0.000654
200065_s_at	ARF1	ADP-ribosylation factor 1	0.000666
210260_s_at	TNFAIP8	tumor necrosis factor, alpha-induced protein 8	0.000669
231629_x_at	KLK3	kallikrein-related peptidase 3	0.000683
201829_at	NET1	neuroepithelial cell transforming 1	0.000719
240633_at	DOK7	docking protein 7	0.000724
225766_s_at	TNPO1	transportin 1	0.00083
219334_s_at	OBFC2A	oligonucleotide/oligosaccharide-binding fold containing 2A	0.000846
227622_at	PCF11	PCF11, cleavage and polyadenylation factor subunit, homolog (S. cerevisiae)	0.000872
217561_at	CALCA	calcitonin-related polypeptide alpha	0.000891
214638_s_at	CCNT2	cyclin T2	0.000898
218624_s_at	MGC2752	hypothetical LOC65996	0.000989

**Table 2:** Cd^2+ ^repressed genes in acute cadmium-exposed human proximal tubule cells (1 day, p value < 0.001)

Probe	Symbol	Gene name	Pr(> |t|)
213067_at	MYH10	myosin, heavy chain 10, non-muscle	4.01E-05
225276_at	GSPT1	G1 to S phase transition 1	8.75E-05
232349_x_at	DCAF6	DDB1 and CUL4 associated factor 6	0.000115
1557986_s_at	SMCR8	Smith-Magenis syndrome chromosome region, candidate 8	0.000121
223141_at	UCK1	uridine-cytidine kinase 1	0.000123
209196_at	WDR46	WD repeat domain 46	0.000125
227477_at	ZMYND19	zinc finger, MYND-type containing 19	0.000149
223396_at	TMEM60	transmembrane protein 60	0.00018
227765_at	NA	NA	0.000189
235812_at	TMEM188	transmembrane protein 188	0.000237
239464_at	NA	NA	0.000239
202337_at	PMF1	polyamine-modulated factor 1	0.000242
1570506_at	NA	NA	0.000247
1555630_a_at	RAB34	RAB34, member RAS oncogene family	0.000249
225831_at	LUZP1	leucine zipper protein 1	0.000254
204027_s_at	METTL1	methyltransferase like 1	0.000295
212694_s_at	PCCB	propionyl Coenzyme A carboxylase, beta polypeptide	0.000304
204104_at	SNAPC2	small nuclear RNA activating complex, polypeptide 2, 45 kDa	0.000369
216308_x_at	GRHPR	glyoxylate reductase/hydroxypyruvate reductase	0.000392
233233_at	NA	NA	0.000438
200980_s_at	PDHA1	pyruvate dehydrogenase (lipoamide) alpha 1	0.000468
218042_at	COPS4	COP9 constitutive photomorphogenic homolog subunit 4 (Arabidopsis)	0.000476
210829_s_at	SSBP2	single-stranded DNA binding protein 2	0.000504
203226_s_at	TSPAN31	tetraspanin 31	0.000516
202266_at	TTRAP	TRAF and TNF receptor associated protein	0.000541
221856_s_at	FAM63A	family with sequence similarity 63, member A	0.00066
227224_at	RALGPS2	Ral GEF with PH domain and SH3 binding motif 2	0.000663
243309_at	FLJ27352	hypothetical LOC145788	0.000673
202883_s_at	PPP2R1B	protein phosphatase 2 (formerly 2A), regulatory subunit A, beta isoform	0.000684
201801_s_at	SLC29A1	solute carrier family 29 (nucleoside transporters), member 1	0.000688
220974_x_at	SFXN3	sideroflexin 3	0.000699
1555679_a_at	RTN4IP1	reticulon 4 interacting protein 1	0.000777
212872_s_at	MED20	mediator complex subunit 20	0.00078
201888_s_at	IL13RA1	interleukin 13 receptor, alpha 1	0.000787
227567_at	NA	NA	0.00079
229146_at	C7orf31	chromosome 7 open reading frame 31	0.000806
224779_s_at	FAM96A	family with sequence similarity 96, member A	0.000843
213022_s_at	UTRN	utrophin	0.000846
226710_at	C8orf82	chromosome 8 open reading frame 82	0.000846
213181_s_at	MOCS1	molybdenum cofactor synthesis 1	0.000878
218268_at	TBC1D15	TBC1 domain family, member 15	0.000883
211708_s_at	SCD	stearoyl-CoA desaturase (delta-9-desaturase)	0.000916
202299_s_at	HBXIP	hepatitis B virus x interacting protein	0.000948
213574_s_at	KPNB1	karyopherin (importin) beta 1	0.00097
205052_at	AUH	AU RNA binding protein/enoyl-Coenzyme A hydratase	0.00097

Our next aim was to identify the gene regulatory pathways that were activated in the HPT cells in response to the acute, 1 day exposure to Cd^2+^. The gene pathway database compiled in Kyoto Encyclopedia of Genes and Genomes (KEGG) has been a valuable source for investigating pathways. The gene set enrichment analysis (GSEA) method was employed to identify activated KEGG pathways using the microarray data. The approach of GSEA is to test whether the ranks of P values of a set of genes are randomly distributed among all the P values [12]. Because most of GSEA methods employ two sample comparison method such as unpaired or paired t test [13], we set out to develop a statistical testing method for GSEA based on regression analysis for each individual gene. As described earlier, out of a total of 28,720 genes, 4036 were found to have a significant P values (< 0.05) correlating to Cd^2+ ^concentrations. Suppose a KEGG pathway has n genes and x genes are significant using regression analysis. Our GSEA method consists of deriving a hypergemetric probability function shown below.

$$p\left(x\right)=\frac{\left(\begin{array}{c} 4036\\ x \end{array}\right)\left(\begin{array}{c} 28720-4036\\ n-x \end{array}\right)}{\left(\begin{array}{c} 22000\\ n \end{array}\right)}.$$

Using this function, we can test whether x is significantly larger than what would be expected from a hypergeometric distribution. Statistically, if we observe r genes in the significant set, P(X > r) designates the P value for the significance of r genes. Thus, P value = P(X > r) = p(r+1) + p(r+2) + p(r+3) + ... +p(x). This gene set enrichment analysis found 3 significant pathways, NOD-like receptor signaling pathway, base excision repair, and steroid biosynthesis, with p values 0.0486, 0.0385, 0.0009, respectively. NOD-like receptors play an important role in regulation of inflammatory and apoptotic responses and are activated to stress response [14]. DNA repair is a mode of action known to response to heavy metal toxicity. Steroid biosynthesis is a metabolic process that is required for recovery from an acute toxicity attack.

### Gene expression variation in response to long term (13 day) cadmium toxicity

HPT cells were exposed to Cd^2+ ^for a long period in order to determine the mechanism by which global gene expression adapted to environmental challenge. HPT cells were exposed to various concentrations of Cd^2+^, 4.5, 9 and 27 μM Cd^+2 ^for 13 days. Lower concentration of Cd^2+ ^were used for the 13 day time course since 45 μM Cd^2+ ^caused toxicity and a loss of cell viability. There was no loss of cell viability when the cells were treated with 4.5 and 9 μM Cd^2+ ^over a 13 day period, while exposure to 27 μM Cd^+2 ^caused an approximate 30% loss in cell viability [10]. The ability of the HPT cells to form domes was only affected by exposure to 27 μM Cd^+2 ^for 13 days. The microarray data were processed and analyzed as described above for 1 day exposure. The distribution of P values for each individual gene is shown in Figure 1b. The distribution is significantly skewed to small P values, implying a large number of genes were altered by cadmium exposure. From a total of 28,720 genes, 366 were positively correlated with the cadmium concentration, and 517 were negatively correlated at a P value cutoff of 0.01 (Table 3 and 4). Similar to that observed for 1 day exposure, long term cadmium exposure altered the expression of a number of cell cycle control and cell death genes, including GSPT1, G2E3 and BCL2. The expression of metal binding and transport genes, such as CAB39L, TMC5, SLC5A3 and SLC35F2, were affected as well. However, expression levels of stress response genes such as heat shock and DNA repair genes were not changed after 13 days exposure, instead, many tumor-related genes were activated. These included RAB42, DEK, RRAD, TPD52L1 and a variety of mitogen related kinases. The GSEA analysis revealed 9 altered KEGG pathways including 3 cancer pathways, prostate cancer, melanoma and p53 signaling pathway (Table 5). None of the pathways overlapped with the ones identified after 24 hours exposure.

**Table 3:** Cd^2+ ^induced genes in chronic cadmium-exposed human proximal tubule cells (13 days, p value < 0.001)

Probe	Symbol	Gene name	Pr(> |t|)
225914_s_at	CAB39L	calcium binding protein 39-like	1.69E-05
210372_s_at	TPD52L1	tumor protein D52-like 1	2.73E-05
211499_s_at	MAPK11	mitogen-activated protein kinase 11	3.07E-05
222587_s_at	GALNT7	UDP-N-acetyl-alpha-D galactosamine:polypeptide N-acetylgalactosaminyltransferase 7 (GalNAc-T7)	6.05E-05
233528_s_at	GATSL3	GATS protein-like 3	8.85E-05
212335_at	GNS	glucosamine (N-acetyl)-6-sulfatase	9.88E-05
217748_at	ADIPOR1	adiponectin receptor 1	0.000112
222601_at	UBA6	ubiquitin-like modifier activating enzyme 6	0.000155
219349_s_at	EXOC2	exocyst complex component 2	0.000168
218319_at	PELI1	pellino homolog 1 (Drosophila)	0.000213
203553_s_at	MAP4K5	mitogen-activated protein kinase kinase kinase kinase 5	0.000219
1560779_a_at	NA	NA	0.000227
244056_at	SFTA2	surfactant associated 2	0.000236
223797_at	PRO2852	hypothetical protein PRO2852	0.000366
219580_s_at	TMC5	transmembrane channel-like 5	0.000373
1553367_a_at	COX6B2	cytochrome c oxidase subunit VIb polypeptide 2 (testis)	0.000413
200618_at	LASP1	LIM and SH3 protein 1	0.000414
203925_at	GCLM	glutamate-cysteine ligase, modifier subunit	0.000502
209198_s_at	SYT11	synaptotagmin XI	0.000509
213107_at	TNIK	TRAF2 and NCK interacting kinase	0.000532
212341_at	YIPF6	Yip1 domain family, member 6	0.000565
223799_at	KIAA1826	KIAA1826	0.000598
228810_at	CCNYL1	cyclin Y-like 1	0.000665
218472_s_at	PELO	pelota homolog (Drosophila)	0.000669
227022_at	GNPDA2	glucosamine-6-phosphate deaminase 2	0.000688
1552712_a_at	NMNAT2	nicotinamide nucleotide adenylyltransferase 2	0.00069
217144_at	UBB	ubiquitin B	0.000699
1559514_at	LOC100132077	hypothetical protein LOC100132077	0.000707
1552946_at	ZNF114	zinc finger protein 114	0.000794
226071_at	ADAMTSL4	ADAMTS-like 4	0.000822
204802_at	RRAD	Ras-related associated with diabetes	0.000874
208816_x_at	ANXA2P2	annexin A2 pseudogene 2	0.00088
212791_at	C1orf216	chromosome 1 open reading frame 216	0.000882
215704_at	FLG	filaggrin	0.000923
200638_s_at	YWHAZ	tyrosine 3-monooxygenase/tryptophan 5-monooxygenase activation protein, zeta polypeptide	0.000945

**Table 4:** Cd^2+ ^repressed genes in chronic cadmium-exposed human proximal tubule cells (13 days, p value < 0.001)

Probe	Symbol	Gene name	Pr(> |t|)
218025_s_at	PECI	peroxisomal D3,D2-enoyl-CoA isomerase	3.66E-05
202781_s_at	INPP5K	inositol polyphosphate-5-phosphatase K	6.81E-05
216053_x_at	FAM182A	family with sequence similarity 182, member A	0.0001
228665_at	CYYR1	cysteine/tyrosine-rich 1	0.000109
228374_at	C10orf28	chromosome 10 open reading frame 28	0.000119
224913_s_at	TIMM50	translocase of inner mitochondrial membrane 50 homolog (S. cerevisiae)	0.000131
215438_x_at	GSPT1	G1 to S phase transition 1	0.000132
218404_at	SNX10	sorting nexin 10	0.000133
226016_at	CD47	CD47 molecule	0.000135
203574_at	NFIL3	nuclear factor, interleukin 3 regulated	0.000136
227190_at	TMEM37	transmembrane protein 37	0.00017
201225_s_at	SRRM1	serine/arginine repetitive matrix 1	0.000211
1552846_s_at	RAB42	RAB42, member RAS oncogene family	0.000217
228520_s_at	APLP2	amyloid beta (A4) precursor-like protein 2	0.000219
227769_at	NA	NA	0.000231
201855_s_at	ATMIN	ATM interactor	0.000256
219379_x_at	ZNF358	zinc finger protein 358	0.000275
32811_at	MYO1C	myosin IC	0.000282
200707_at	PRKCSH	protein kinase C substrate 80K-H	0.000296
229007_at	LOC283788	FSHD region gene 1 pseudogene	0.000301
219271_at	GALNT14	UDP-N-acetyl-alpha-D-galactosamine:polypeptide Nacetylgalactosaminyltransferase 14 (GalNAc-T14)	0.000304
201816_s_at	GBAS	glioblastoma amplified sequence	0.000305
217817_at	ARPC4	actin related protein 2/3 complex, subunit 4, 20 kDa	0.000315
201185_at	HTRA1	HtrA serine peptidase 1	0.000316
205739_x_at	ZNF107	zinc finger protein 107	0.000323
212418_at	ELF1	E74-like factor 1 (ets domain transcription factor)	0.000327
209431_s_at	PATZ1	POZ (BTB) and AT hook containing zinc finger 1	0.000367
201549_x_at	KDM5B	lysine (K)-specific demethylase 5B	0.000373
203858_s_at	COX10	COX10 homolog, cytochrome c oxidase assembly protein, heme A: farnesyltransferase (yeast)	0.000425
218483_s_at	C11orf60	chromosome 11 open reading frame 60	0.00044
218045_x_at	PTMS	parathymosin	0.000454
212493_s_at	SETD2	SET domain containing 2	0.000459
204805_s_at	H1FX	H1 histone family, member X	0.000471
226926_at	DMKN	dermokine	0.000474
211025_x_at	COX5B	cytochrome c oxidase subunit Vb	0.000494
212904_at	LRRC47	leucine rich repeat containing 47	0.000544
215543_s_at	LARGE	like-glycosyltransferase	0.000546
228998_at	TNRC6B	trinucleotide repeat containing 6B	0.000575
210220_at	FZD2	frizzled homolog 2 (Drosophila)	0.000632
225247_at	C19orf6	chromosome 19 open reading frame 6	0.00065
212264_s_at	WAPAL	wings apart-like homolog (Drosophila)	0.00066
244287_at	SFRS12	splicing factor, arginine/serine-rich 12	0.000661
221829_s_at	TNPO1	transportin 1	0.000704
1553313_s_at	SLC5A3	solute carrier family 5 (sodium/myo-inositol cotransporter), member 3	0.000786
232412_at	FBXL20	F-box and leucine-rich repeat protein 20	0.000788
217964_at	TTC19	tetratricopeptide repeat domain 19	0.000788
223056_s_at	XPO5	exportin 5	0.000792
213470_s_at	HNRNPH1	heterogeneous nuclear ribonucleoprotein H1 (H)	0.000816
214543_x_at	QKI	quaking homolog, KH domain RNA binding (mouse)	0.00085
243829_at	BRAF	v-raf murine sarcoma viral oncogene homolog B1	0.000864
34260_at	TELO2	TEL2, telomere maintenance 2, homolog (S. cerevisiae)	0.000874
203392_s_at	CTBP1	C-terminal binding protein 1	0.000877
201928_at	PKP4	plakophilin 4	0.000888
232814_x_at	KLC1	kinesin light chain 1	0.000907
212114_at	LOC552889	hypothetical protein LOC552889	0.000922
225399_at	TSEN15	tRNA splicing endonuclease 15 homolog (S. cerevisiae)	0.000924
236035_at	NA	NA	0.000931
229400_at	HOXD10	homeobox D10	0.000961
208847_s_at	ADH5	alcohol dehydrogenase 5 (class III), chi polypeptide	0.000984
214800_x_at	BTF3	basic transcription factor 3	0.000992
204619_s_at	VCAN	versican	0.000997
218826_at	SLC35F2	solute carrier family 35, member F2	0.000998

**Table 5:** Cd^2+ ^activated KEGG pathways in chronic cadmium-exposed human proximal tubule cells (13 days)

KEGG Pathway	P value
Lysine biosynthesis	0.000182016
Sulfur metabolism	0.001924676
Glutathione metabolism	0.001973874
Caffeine metabolism	0.004896048
Protein processing in endoplasmic reticulum	0.022267231
Prostate cancer	0.035237612
Melanoma	0.035316953
Valine, leucine and isoleucine degradation	0.038038497
p53 signaling pathway	0.041271084

### The common expression patterns between 1 day and 13 day of cadmium exposure

**Figure 2:**
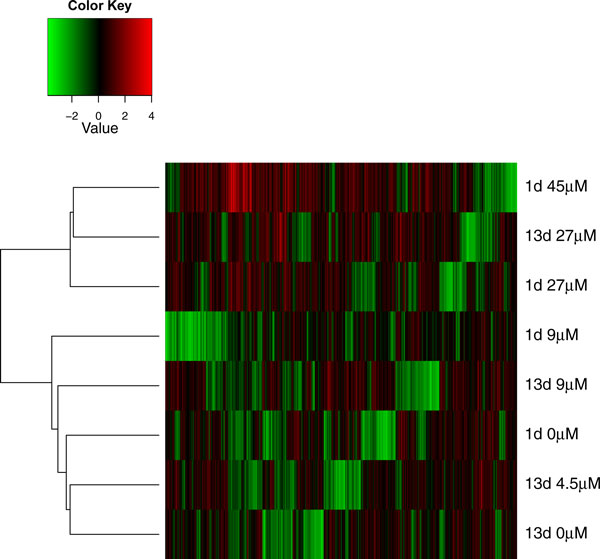
**The heat map of global gene expression for all 8 HPT cell samples**. Each row represents a HPT cell sample, and each column represents a gene. Only the top 901 genes that have high variability across 8 samples are plotted in the heat map. A dendrogram of hierarchical clustering is shown on the left side of the map.

**Figure 3:**
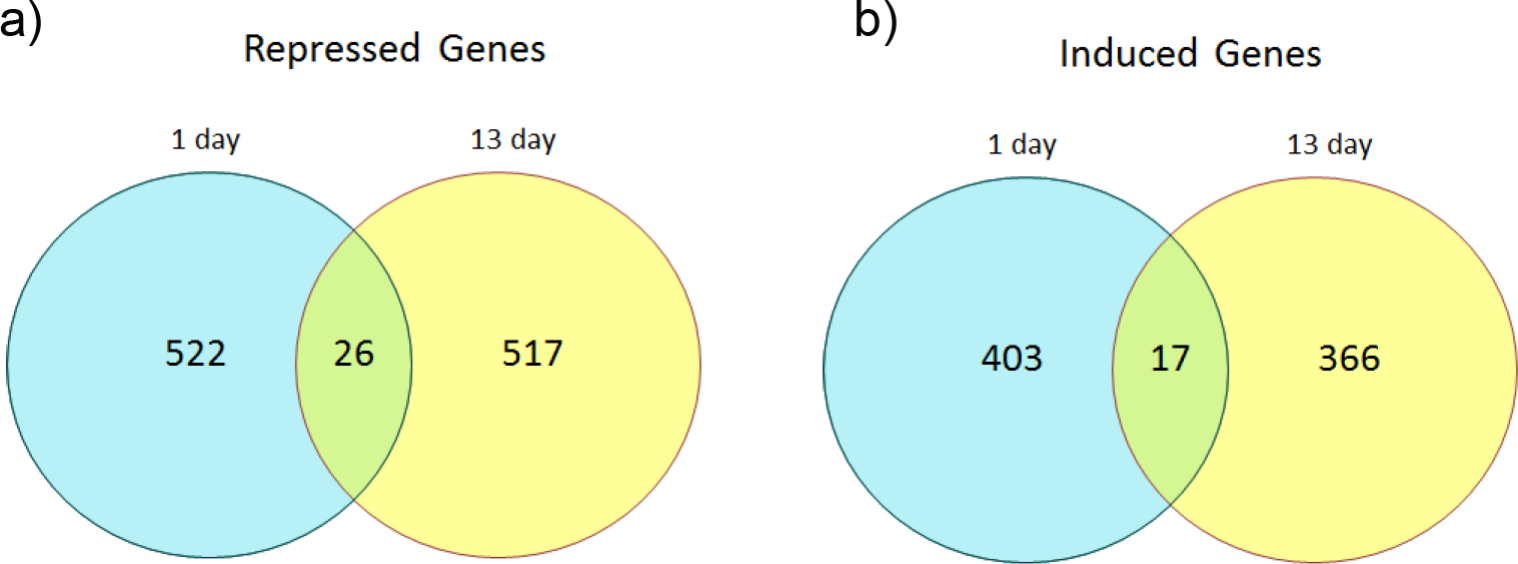
**Venn diagram depicting the number of genes activated in each treatment group**. Shown are cadmium correlated genes specific for acute (24 h) and chronic (13 days) exposure and genes common to both treatment groups. The number of cadmium correlated genes in each group is also broken down into the number of genes induced or repressed by cadmium.

In order to investigate genes that showed expression variation throughout both short and long term cadmium exposure, we conducted analysis for the pooled data of the two time points. Figure 2 shows a heat map for the 8 samples for 1 day and 13 day exposure. The data were normalized to a grand mean of 0 and only 901 genes with standard deviation > 1 were used for this analysis. Hierarchical clustering method that was based on Pearson dissimilarity and Ward linkage showed as expected that the HPT cells treated with high doses of Cd^2+ ^tended to be grouped together. The cells exposed to 27 and 45 μM Cd^2+ ^for both 1 day and 13 days formed a group, and all cells exposed to 9 μM or lower Cd^2+ ^concentration formed the other major group. The change of dissimilarity and linkage methods did not alter the results. There were 17 genes that were positively correlated with cadmium concentration for both 1 day and 13 day exposure (Figure 3a). Given that the number of positively correlated genes was 403 and 366 for 1 and 13 day respectively, the random number of overlapping genes can be modeled as a hypergeometric distribution. The probability of observing more than 17 overlapping genes from the random sets of 403 and 366 genes is 5.89E-06. Thus, the two exposure time periods had a significant number of common genes that were positively correlated with cadmium concentration. Similarly, we found 26 genes that were negative correlated with cadmium concentration for both time periods (Figure 3b). This number of overlapping genes was statistically significant when compared to selecting 26 gene by chance (P value = 1.32E-06). Therefore, we conclude that the HPT cells have a common response component in global gene expression variation during short term and long term cadmium exposure.

### Gene regulatory network in response to cadmium exposure

**Figure 4:**
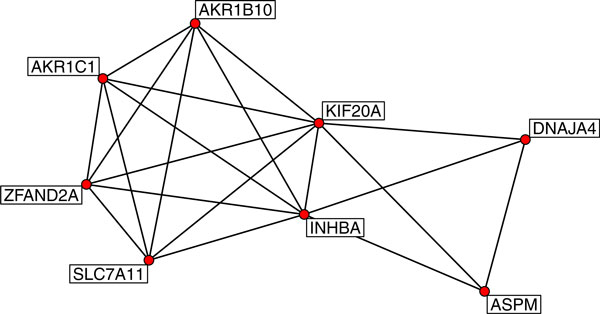
**A network module of 8 genes that interact during cadmium toxicity**. The edge between two genes indicates they are interacting directly.

In response to heavy metal toxicity, thousands of genes will be activated forming a complicated gene regulatory network interacting cooperatively. To decode the complete gene network for cadmium response is impossible for the small sample size used in this study and high complexity of network structure [15]. It is also computational forbidden to construct a network by including a large number of genes. Therefore, we modeled the gene network focusing on only the 901 identified genes as having high variability shown in the heat map. Mutual information for transcriptional levels was used to quantify the relations between genes. The resulting adjacent matrix was analyzed for the presence of an active network module that has high connectivity within the module and low connectivity with other components of the network. Figure 4 shows a network modules consisting of 8 genes. DNAJA4 encodes the heat shock protein, SLC7A11 an anion amino acid transporter, AKR1B10 and AKR1C1 are subunits for a reductase associated with cancer, ASPM and KIF20A are involved in mitotic spindle functions, ZFAND2A is a zinc-finger protein that is inducible by arsenite, and INHBA is known to inhibit cell proliferation and to have tumor-suppressor activity. The network modules provide hypotheses for gene interactions which can be verified in subsequent biological experiments.

## Discussion

Microarray experiments have become a standard approach to simultaneously quantify the transcriptional levels of thousands genes under various conditions. However, due to the high cost of chips, microarray experiments are often carried out with a limited sample size, which requires a careful experimental design. In this study, we chose two time points, 1 day and 13 day, to account for toxicity caused by short and long cadmium exposure. For each time point, we had HPT cells exposed to 3 cadmium concentrations plus a control sample (non-treated). Thus, even with only a single replicate at each concentration, we were able to test the correlation between transcriptional levels and cadmium concentrations. Our analysis suggests that experimental design with various conditions overcomes the difficulty of lack of replications.

Exposure of HPT cells to 1 day and 13 days of cadmium induces distinct sets of genes. At 1 day, HPT cells appeared to have a stress response that activated a number of heat shock and DNA repair genes and pathways. This was expected because stress responses that are characterized with induced expression of heat shock proteins are acute and transient that last for only a few hours to a few days [16-20]. This acute response to Cd^2+ ^with the induction of heat shock proteins, such as HSP 27, 60, 70 and 90, have been repeatedly verified in our previous studies [21-24].

The 13 days exposure was used to model the chronic response of kidney to environment of cadmium exposure. The molecular changes in the HPT cells in response to long term cadmium exposure were manifested with activation of many oncogenes and cancer pathways. The association of cancer occurrence and cadmium exposure has been established by numerous cohort studies [25-27]. Although 13 days of exposure might not exactly represent the chronic effect of cadmium on the kidney living environment, our experiment suggests that the cell culture system serves as a valid model for studying the long term toxicity of cadmium on the kidney.

Although the two time periods of cadmium exposure appeared to induce two distinct toxicity responses, the acute and chronic responses, they shared some common features in gene expression. The heat map showed that the samples were not separated by time of exposure, instead, the samples tended to be grouped by doses across two time points. All the HPT samples that were treated with 27 μM Cd^2+ ^for both 1 day and 13 days formed a cluster. When looking into the genes that were correlated with cadmium concentrations in both 1 day and 13 days, we found that the number of overlapping genes was significantly larger than what was expected from random selection. The set of overlapping genes included ion transporter proteins, kinases, and transcriptional factors. These genes should be further investigated to reveal their functions in toxicity responses.

For any biophysical process, thousands of genes interact to form a network to maintain their biological functions. There are commonly two statistical approaches to study gene interaction, gene set enrichment analysis (GSEA) and networks analysis. GSEA has been successfully applied to microarray data to identify the activated pathways. However, GSEA has been so far focused on comparing different treatment groups, mostly for two groups, treatment and control [12,13,28,29]. For this study, we developed a GSEA method that can be used for ranks of P values from any statistical tests. We have showed that our proposed GSEA statistic follows a hypergeometric distribution. Thus, the P value of the GSEA is available.

Gene networks analysis is computationally challenging. Networks are often highly complicated, consisting of a large number of genes that can be fairly dynamic in nature. Decoding such a gene network requires researchers to quantify the global gene expression under dynamic and variable conditions. The current study provides a unique opportunity in that microarray data was acquired at multiple time points and at various levels of Cd^2+ ^exposure. We used mutual information to account for the relations between genes. Mutual information is able to measure gene dependency without assuming data distributions and linear relationship. However, the mutual information-based network models require huge amount of computation that is not feasible for modeling all the genes. For this reason, we focused on only a set of most variable genes across all samples. The network analysis revealed gene network modules consisting of a small group of genes that intensively interacted with each other. The network modules can be verified in molecular experiments and they serve as hypothesized models for functional relations of genes when cooperating for toxicology responses induced by cadmium.

## Competing interests

The authors declare that they have no competing interests.

## Authors' contributions

SG, SS and DS designed and conducted the experiments. KC carried out regression analysis. YD contributed to network analysis. KZ contrived the analysis plan and developed analysis tools. KZ and SG contributed in writing the manuscript.
